# High prevalence of non-communicable diseases and associated risk factors amongst adults living with HIV in Cambodia

**DOI:** 10.1371/journal.pone.0187591

**Published:** 2017-11-09

**Authors:** Pheak Chhoun, Sovannary Tuot, Anthony D. Harries, Nang Thu Thu Kyaw, Khuondyla Pal, Phalkun Mun, Carrine Brody, Gitau Mburu, Siyan Yi

**Affiliations:** 1 KHANA Center for Population Health Research, Phnom Penh, Cambodia; 2 International Union against Tuberculosis and Lung Disease, Paris, France; 3 London School of Hygiene and Tropical Medicine, London, United Kingdom; 4 International Union Against Tuberculosis and Lung Disease, Myanmar Country Office, Mandalay, Myanmar; 5 Surveillance Unit, National Center for HIV/AIDS, Dermatology, and STDs, Phnom Penh, Cambodia; 6 Center for Global Health Research, Touro University, California, United States of America; 7 Division of Health Research, Lancaster University, Lancaster, United Kingdom; Azienda Ospedaliera Universitaria di Perugia, ITALY

## Abstract

**Background:**

With rapid expansion of antiretroviral therapy for HIV, there are rising life expectancies among people living with HIV. As a result, co-morbidity from non-communicable diseases in those living and aging with HIV is increasingly being reported. Published data on this issue have been limited in Cambodia. The aim of this study was to determine the prevalence of diabetes mellitus, hypertension and hypercholesterolemia and associated risk factors in adults living with HIV in Cambodia.

**Methods:**

This cross-sectional study was conducted in five provinces of Cambodia from May-June 2015. Information was obtained on socio-demographic and clinical characteristics through face-to-face interviews using a structured questionnaire, and anthropometric and biochemical measurements were performed. Diabetes mellitus was diagnosed with fasting blood glucose ≥126 mg/dl, hypertension with systolic blood pressure ≥140 mmHg and/or diastolic blood pressure ≥ 90 mmHg and hypercholesterolemia with fasting blood cholesterol ≥190 mg/dl. Multivariable logistic regression analyses were used to explore risk factors.

**Results:**

The study sample included 510 adults living with HIV; 67% were female, with a mean age of 45 (standard deviation = 8) years. Of these, 8.8% had diabetes mellitus, 15.1% had hypertension and 34.7% had hypercholesterolemia. Of the total participants with non-communicable diseases (*n* = 244), 47.8% had one or more diseases, and 75% were not aware of their diseases prior to the study: new disease was diagnosed in 90% of diabetes mellitus, 44% of hypertension and 90% of hypercholesterolemia. Single disease occurred in 81%, dual disease in 17% and triple disease in 2%. In adjusted analyses, those consuming 1 serving of fruit compare to 2 servings as significantly with diabetes mellitus, those eating 1 serving of fruit compare to 2 servings and using lard for cooking were significantly associated with hypertension, and those being unemployed, having monthly income less than 100 USD and being underweighted were significantly associated with hypercholesterolemia.

**Conclusions:**

The prevalence of diabetes mellitus, hypertension and hypercholesterolemia in adults living with HIV in this study was considerably high, with most of these diseases newly identified through active screening in the survey. These findings strongly suggest that screening of non-communicable diseases should be integrated into routine HIV care in Cambodia.

## Introduction

In recent years, there has been a rapid expansion of antiretroviral therapy (ART) for people living with HIV. Of the 36.7 million people living with HIV globally at the end of 2015, 17 million (46%) were estimated to be receiving ART [1]. The scale up of ART for people living with HIV in low- and middle-income countries has been associated with huge individual benefits and rising life expectancies [2–4].

However, the expanded access to ART has also resulted in a new global health challenge, namely, increased co-morbidity of non-communicable diseases in those living and aging with HIV [5, 6]. It is well established that cardiovascular disease, hypertension and diabetes mellitus all have direct and indirect relationships with HIV and ART, and these diseases are also a growing problem in the general population living in low- and middle-income countries, due to urbanization, changes in diet and lifestyle, and increases in life expectancy [7].

Several reasons for an excess of these non-communicable diseases in adults living with HIV have been reported. First, similar to the general populations, people living with HIV often have traditional risk factors for non-communicable diseases such as smoking and alcohol [8]. Second, certain ART drugs contribute to these complications through hypercholesterolemia, increased abdominal fat and the metabolic syndrome, although toxicity has decreased among newer generations of antiretroviral drugs [5]. Third, in people living with HIV, whether they have been initiated on ART or not, there is chronic activation of the innate immune system with excessive production of inflammatory cytokines and mediators that in turn are associated with an increased risk of atherosclerosis, coronary artery inflammation and all-cause mortality [5, 8, 9].

Consequently, the body of evidence linking HIV with non-communicable diseases has grown over the last decade in both high-income and low- and middle-income countries. Several studies in sub-Saharan African countries including Nigeria, Tanzania, Malawi and Botswana have found significant rates of diabetes mellitus and hypertension in people living with HIV on ART, with cumulative exposure to ART being significantly associated with the development of diabetes mellitus [10–13]. However, published evidence about the prevalence or risk of non-communicable diseases among people living with HIV in Asia has been limited [14].

In Cambodia, the estimated HIV prevalence in the general population aged 15 to 49 is 0.6% in 2014 [15], which is concentrated among key populations including men who have sex with men (2.2%), transgender women (5.9%), female entertainment workers (9.8%) and injecting drug users (24.8%) [16–19]. Coverage of ART is high, with 96% of people living HIV in need for ART currently receiving it [20]. Prevalence of diabetes mellitus, hypertension and hypercholesterolemia in the general adult population in Cambodia is estimated to be approximately 5%, 11% and 21%, respectively [21, 22]. However, no published data about the prevalence of these diseases among people living with HIV have been reported, and no programmes that routinely screen for non-communicable diseases among people living with HIV has been implemented. Information on non-communicable diseases among this vulnerable population in the country is important as it can inform health policy strategies and appropriate clinical interventions for better management of the overlapping epidemics of HIV and non-communicable diseases with a view to reducing co-morbidity and mortality [23]. The aim of this study was to determine the prevalence of diabetes mellitus, hypertension and hypercholesterolemia in adults living with HIV in Cambodia, and explore the demographic, clinical and anthropometric factors associated with these three diseases.

## Materials and methods

### Ethical statement

The study was approved by the National Ethics Committee for Health Research (NECHR—Ref number 119NECHR / 27/04/2015) in Cambodia, the Ethical Committee of Touro University California, the United States, and the Ethics Advisory Group of the International Union Against Tuberculosis and Lung Disease, Paris, France. The study was conducted in full compliance with the protocol, and was not amended.

In compliance with consent and confidentiality, individuals were informed that their participation in the study was voluntary, both before and after consenting, and they were asked to provide written informed consent. The survey was anonymous and used unique participant identification numbers. No personal identifiers were collected. The questionnaires and data collected from the respondents were kept in secure cabinets and password protected computers at the KHANA Center for Population Health Research.

After assessment, each participant was informed about and given a hard copy of the results of the physical assessments and biochemical tests, and also given appropriate health education. If the study participant was found to have either a non-communicable disease or one or more risk factors, the information was communicated to the study coordinator for further counseling, evaluation, assessment, management and monitoring.

### Design and setting

This was a cross-sectional study using a structured questionnaire in adults living with HIV. The study was conducted in Cambodia, which is a Southeast Asian country bordered by Thailand, Laos and Vietnam. The country has a population of 15 million and a gross national income of USD$ 2,330 per capita [24]. Life expectancy of both men and women in 2012 was 72 years, having increased from 54 years in 1999 [24]. The study was conducted by KHANA Center of Population Health Research. KHANA is the largest national non-governmental organization (NGO) providing integrated HIV prevention, care and support services for people living HIV at the community level through 19 local NGOs and networks in 23 of the country’s 25 municipality and provinces. Currently, about 20,000 (35%) people living with HIV out of 57,000 known to be in care in Cambodia are receiving care under KHANA. The care received includes ART, which became available in Cambodia to people living with HIV in 2001. Criteria for starting ART have changed over time, but in 2015 (at the time of the study) people living with HIV were eligible for ART if they were in WHO clinical stage 3 or 4, or if they had a CD4 cell count < 350 cells/uL.

### Participants and sample size

Participants were adults living with HIV who were (1) aged ≥ 21 years; (2) known to have HIV infection for at least 12 months; (3) willing to provide consent and (4) able to attend at the selected health centers on the day of data collection. In addition, for those on ART, participants had to have been on the treatment for at least six months. Pregnant women and breast-feeding mothers were excluded from the study. At the time of the study, there were 75,000 people estimated to be living with HIV nationally [15, 25]. Assuming a prevalence of non-communicable diseases of 50% and a precision of 5%, the sample size was calculated to be 383. To account for 20% non-responders, at least 460 participants were required for the study. Study participants were interviewed and assessed between May and June 2015.

### Sampling and recruitment

The capital city of Phnom Penh and four provinces of Battambang, Pursat, Siem Reap, and Takeo were purposively selected out of 25 municipality and provinces in the country. We determined that more than 70% of the people living with HIV covered by KHANA’s activities resided in these four provinces and a city. A two-stage cluster sampling method was used to select the study sample. In the first stage, we made a list of health centers (*n* = 65) with at least 20 adults living with HIV in the catchment areas. This list formed a sampling frame from which 34 health centers were randomly selected. In the second stage, 15 participants from each of those health centers who met the study inclusion criteria were selected by picking every odd-numbered participant from the eligible participant list. If the number of the odd-numbered participants was not sufficient at any clinic, further selection was made by choosing the even-numbered participants. Once identified, the participants were invited by field coordinators to participate in the study, and appointments were made for them to come to the health centers for questionnaire administration, anthropometric measurements and the drawing of blood samples.

### Measures and definitions

Through the questionnaire, information was collected on socio-demographic characteristics, established risk factors for non-communicable disease and clinical parameters. Anthropometric and biochemical measurements were performed at the same time with data entered into the questionnaire. Socio-demographic variables included age, sex, education level, occupation, monthly income (USD), tobacco use, alcohol consumption, fruit and vegetable servings, type of oil used for cooking and physical activity. Clinical parameters included time since HIV diagnosis, time since ART was initiated, type of ART especially with a protease inhibitor, a previous history of diabetes mellitus, hypertension or hypercholesterolemia and lifestyle advice received in the last three years from a health care provider. Anthropometric measurements included weight, height, a derived body mass index (BMI = weight in kg/ height in m^2^) and waist circumference (in cm). Biomedical measurements and samples collected included systolic blood pressure (in mmHg), diastolic blood pressure (in mmHg), fasting blood glucose (in mg/dl) and fasting blood cholesterol (in mg/dl). Blood glucose and cholesterol were both measured using finger prick and portable equipment, the instruments being regularly calibrated during the study period (EasySure GCU, CEI Technology Inc., Taiwan). Definitions of terms and variables are shown in Table 1 which are consistent with exiting guidelines and previous studies [22, 26]. For the purpose of the current study, diabetes mellitus, hypertension and hypercholesterolemia were collectively referred to as “non-communicable diseases”.

**Table 1:** Definitions of terms and variables used in the study.

Term or variable	Definition
One drink or one standard drink	10 grams of pure alcohol consumption across different alcoholic beverages. Approximately 1 bottle/can/pot (330 ml) of beer or fermented palm juice alcohol with 4–5% ethanol. Show cards were used to illustrate these quantities
One serving of fruits or vegetables	80 grams of fruit & vegetables. Approximately 1 medium size piece of fresh fruit, a glass of fruit juice, 3–5 pieces of small size fruits, half cup of dried fruit, a slice of water melon, a cup of raw vegetables, and half a cup of steamed vegetables. Show cards were used to illustrate these quantities.
Physical activity in leisure time High Moderate Low	High = vigorous activity at least 3 days per week of at least 150 minutes per week or moderate activity on seven days. Moderate = vigorous activity on 3 days per week of at least 20 minutes per day or five days of moderate activity of walking of at least 30 minutes per day. Low = not high or moderate
BMI cut-off levels Underweight Normal Overweight Obese	BMI<18.5 kg/m^2^ BMI 18.5–22.99 kg/m^2^ BMI 23–27.49 kg/m^2^ BMI>27.50 kg/m^2^
Abdominal obesity	Waist circumference >90 cm (M) and > 80 cm (F)
Fasting capillary plasma glucose Normal or impaired Diabetes mellitus	< 126 mg/dl) ≥ 126 mg/ dl
Total blood cholesterol Normal Hypercholesterolemia	<190mg/dL ≥190 mg/ dl
Blood pressure Normal Hypertension	SBP <140 and /or DBP <90 * SBP ≥140 and/ or DBP ≥ 90 *

*Systolic and diastolic blood pressure was calculated as the mean of the second and the third blood pressure measurement

Abbreviation: BMI = body mass index; BP = blood pressure; DBP = diastolic blood pressure; F = female; M = male; SBP = systolic blood pressure;

### The questionnaire and data collection

The questionnaire for face-to-face interviews was developed and modified from the structured questionnaire used by the WHO STEPwise approach to non-communicable disease risk factor surveillance (STEPS) [27]. The questionnaire was pre-tested before utilization (S1 Tool). An additional section on history of ART use was added using validated questions developed by the AIDS Clinical Trials Group (ACTG) [28]. The data collection was conducted by five trained data collectors who administered the questionnaire and one trained nurse from each health center who collected information on clinical parameters, performed anthropometric measurements and took blood samples for biomedical measurements. Blood pressure was measured in mmHg (systolic & diastolic) using an automatic blood pressure monitor by Microlife, model BP 3AQ1. Blood pressure was measured three times to ensure the accuracy of the readings, with the mean of the second and third readings being used for the final result. Interviews to administer the questionnaire were performed in a private room located in the data collection sites within the health centers. Travel stipends were given to participants to reimburse their transport costs.

### Data analyses

The collected data were coded, double-entered and analysed in EpiData (version 3.1 for data entry and version 2.2 for data analyses, EpiData Association, Odense, Denmark). STATA Version 12 for Windows (Stata Corp, Texas, USA) was used for data analyses. Characteristics of participants and the frequencies of non-communicable diseases were summarized. Exposure variables were compared against the outcomes of interest (diabetes mellitus and/or hypertension and/or hypercholesterolemia) using Chi-square test with odds ratios (OR) and 95% confidence intervals (CI). Any characteristic associated with an outcome that had a *P* value of ≤0.2 in bivariate analyses was inputted into a multivariable logistic regression model to explore risk factors of non-communicable diseases. A two-sided *P*-value of less than 0.05 was regarded as statistically significant.

## Results

This study included 510 adults living with HIV, of whom 340 (67%) were female, with a mean (standard deviation, SD) age of 45 (8) years. Of the total sample, 8% had diabetes mellitus, 15% had hypertension and 35% had hypercholesterolemia (Table 2). Of the total, 244 patients (48%) were with one or more non-communicable diseases, and 78% of participants with these diseases were not aware of these conditions prior to the study. New disease was diagnosed in 90% of those with diabetes mellitus, 44% of those with hypertension and 90% of those with hypercholesterolemia (Table 2).

**Table 2:** Numbers and proportions of selected non-communicable diseases in 510 adults living with HIV in Cambodia and mean and standard deviations for fasting blood glucose, systolic and diastolic blood pressure and blood cholesterol in patients with newly diagnosed and previously known disease.

**A: Numbers and proportions of selected non-communicable diseases**	New*		Known**		Total***	
	**N**	**(%)**	**n**	**(%)**	**n**	**(%)**
Diabetes mellitus	38	(7.5)	5	(1.0)	43	(8.4)
Hypertension	34	(6.7)	43	(8.4)	77	(15.1)
Hypercholesterolemia	160	(31.4)	17	(3.3)	177	(34.7)
**B: Mean and standard deviations for fasting blood glucose, systolic and diastolic blood pressure and blood cholesterol in patients with newly diagnosis and known disease**	**Mean**	**SD**	**Mean**	**SD**	**Mean**	**SD**
Diabetes mellitus (fasting blood glucose in mg/dl)	167.1	37.6	144.6	30.9	164.5	37.2
Hypertension (systolic blood pressure in mmHg)	181	24.3	176.2	19	178.3	21.5
Hypertension (diastolic blood pressure in mmHg)	113.8	15.2	109.1	12.9	111.2	14.1
Hypercholesterolemia (blood cholesterol in mg/dl)	212	25.8	156.9	37.2	206.7	36.5

* New refers to cases detected as a result of measurements made during the study

** Known refers to cases reported as previously diagnosed and / or on treatment

*** Total refers to all cases (new and known together)

Mean values with SDs for fasting blood glucose, systolic and diastolic blood pressure and hypercholesterolemia in patients with new and previously known diabetes mellitus, hypertension and hypercholesterolemia are shown in Table 2. Lower level of fasting blood glucose and cholesterol as well as systolic and diastolic blood pressure were found in patients with already known disease. New patients were identified as a result of the study and were not on any treatment.

The numbers and proportions of all adults with one disease, two diseases or three diseases combined are shown in Table 3. Of the participants with non-communicable diseases, single disease occurred in 81%, dual disease in 17% and triple disease in 2%.

**Table 3:** Numbers and proportions of selected non-communicable diseases (single or combined) in 510 adults living with HIV in Cambodia.

Selected non-communicable diseases	n	(%)
Diabetes mellitus alone	19	(3.7)
Hypertension alone	47	(9.2)
Hypercholesterolemia alone	132	(25.9)
Diabetes mellitus and hypertension	1	(0.2)
Diabetes mellitus and hypercholesterolemia	16	(3.1)
Hypertension and hypercholesterolemia	24	(4.7)
Diabetes mellitus and hypertension and hypercholesterolemia	5	(1.0)

Demographic, clinical and anthropometric characteristics associated with any non-communicable disease are shown in Table 4. In bivariate analyses, living in an urban community, having an income 1–50 USD per month, experienced in smoking, having one vegetable serving per day compared with two or more servings per day and using lard for cooking, having overweight were associated with a significantly higher risk of non-communicable disease. Having low level of physical activity and underweight were associated with a significantly lower risk of non-communicable disease. All factors remained significant in the adjusted analysis except having low level of physical activity during leisure time.

**Table 4:** Associations between demographic, clinical and anthropometric characteristics and having one or more non-communicable diseases in 510 adults living with HIV in Cambodia.

Characteristics at evaluation		Total	One or more non-communicable disease		OR	(95% CI)	*P* value	aOR	(95% CI)	*P* value
		n	n	(%)						
Age group in years:										
	22–30	14	7	(50.0)	ref					
	31–40	150	56	(37.3)	0.6	(0.2–1.8)	0.35			
	41–50	198	96	(48.5)	0.9	(0.3–2.8)	0.91			
	51-Higher	148	85	(57.4)	1.4	(0.5–4.0)	0.59			
Sex:										
	Male	170	90	(52.9)	ref					
	Female	340	154	(45.3)	0.7	(0.5–1.1)	0.10	0.9	(0.6–1.7)	0.94
Type of community:										
	Rural	202	72	(35.6)	ref					
	Urban	308	172	(55.8)	2.3	(1.6–3.3)	<0.001	2.0	(1.3–3.0)	0.001
Level of education:										
	None	115	53	46.1	0.9	(0.6–1.4)	0.66			
	Any attendance at school	395	191	(48.4)	ref					
Occupation:										
	Unemployed	124	61	(49.2)	1.2	(0.8–1.9)	0.34	0.2	(0.2–1.4)	0.21
	Manual work	221	97	(43.9)	ref					
	Office work	165	86	(52.1)	1.4	(0.9–2.1)	0.10	1.2	(0.8–1.9)	0.37
Monthly income in past year ($):										
	None	111	59	(53.2)	ref					
	1–50	170	67	(39.4)	0.6	(0.4–0.9)	0.02	0.3	(0.1–0.7)	0.003
	51–100	110	53	(48.2)	0.8	(0.5–1.4)	0.45	0.3	(0.1–0.8)	0.01
	> 100	119	65	(54.6)	1.1	(0.6–1.8)	0.82	0.4	(0.1–0.9)	0.03
Tobacco use:										
	Never smoked	377	171	(45.4)	ref					
	Ex-smoker	58	38	(65.5)	2.3	(1.3–4.1)	0.004	2.8	(1.2–5.3)	0.01
	Current smoker	75	35	(46.7)	1.0	(0.6–1.7)	0.83	1.4	(0.6–2.7)	0.38
Alcohol consumption:										
	Never	231	106	(45.9)	ref					
	Ex-drinker	66	29	(43.9)	0.9	(0.5–1.6)	0.77			
	Current drinker	213	109	(51.5)	1.2	(0.9–1.8)	0.26			
Fruit servings per day:										
	None	84	36	(42.9)	0.7	(0.4–1.3)	0.27			
	1 serving	277	133	(48.0)	0.9	(0.6–1.4)	0.64			
	2 servings or more	149	75	(50.3)	ref					
Vegetable servings per day:										
	1 serving	57	38	(66.3)	2.4	(1.3–4.3)	0.002	2.3	(1.2–4.4)	0.008
	2 serving or more	453	206	(45.5)	ref					
Oil type used for cooking:										
	None	2	2	(100.0)	-	-	-	-	-	-
	Lard	22	16	(72.7)	3.1	(1.9–7.9)	0.01	3.4	(1.2–9.7)	0.01
	Vegetable oil	486	226	(46.5)	ref					
Physical activity in leisure time:										
	Low	249	103	(41.4)	0.6	(0.4–0.9)	0.008	0.8	(0.5–1.2)	0.24
	Moderate	25	15	(60.0)	1.3	(0.6–3.0)	0.52	1.2	(0.5–2.9)	0.71
	High	236	126	(53.4)	ref					
Lifestyle advice from health- worker:										
	No	263	118	(44.9)	1.3	(0.9–1.8)	0.16	0.8	(0.5–1.1)	0.18
	Yes	247	126	(51.0)	ref					
Time since HIV diagnosis in months:										
	12–24	17	5	(29.4)	ref					
	25 and above	493	239	(48.5)	2.3	(0.8–6.5)	0.12	2.1	(0.7–6.5)	0.2
ART status:										
	Not on ART	17	7	(41.2)	ref					
	On ART	493	237	(48.1)	1.3	(0.5–3.5)	0.57			
Length on ART in months (n = 493):										
	6–12	2	1	(50.0)	ref					
	13–60	121	53	(43.8)	0.8	(0.1–12.7)	0.86			
	61 and above	358	180	(50.3)	1.0	(0.1–16.3)	0.99			
Type of ART Regimen (n = 493):										
	ART with PI	35	18	(51.4)	1.2	(0.6–2.3)	0.68			
	ART without PI	458	219	(47.8)	Ref					
Weight (BMI) at evaluation:		510								
	Underweight	99	31	(31.3)	0.5	(0.3–0.8)	0.003	0.5	(0.3–0.9)	0.01
	Normal	302	145	(48.0)	ref					
	Overweight	88	55	(62.5)	1.8	(1.1–2.9)	0.01	1.9	(1.1–3.5)	0.02
	Obese	21	13	(61.9)	1.8	(0.7–4.4)	0.21	2.1	(0.7–6.1)	0.16
Abdominal obesity		510								
	Obese	99	53	(53.5)	1.3	(0.8–2.1)	0.20	0.9	(0.5–1.6)	0.76
	Non-obese	411	191	(46.5)	ref					

Abbreviation: aOR = adjusted odds ratio; ART = antiretroviral therapy; BMI = body mass index; CI = confidence interval; OR = odds ratio; PI = protease inhibitor

Characteristics independently associated with a significantly increased risk of diabetes mellitus on its own, hypertension on its own and hypercholesterolemia on its own, were analyzed and the data are shown in three tables presented in S1, S2 and S3 Tables respectively. After adjusting, the principal findings in brief were as follows. Consuming 1 serving of fruit compare to 2 serving (aOR 0.3, 95% CI 0.1–0.8, *P* = 0.01) was significantly associated with diabetes mellitus (S1 Table), Living in an urban environment (aOR 19.0, 95% CI 5.5–65.0, *P*<0.001), eating 1 serving of fruit compare to 2 serving (aOR 0.5, 95% CI 0.3–1.0, *P* = 0.02) and using lard (aOR 4.8, 95% CI 1.4–16.5, *P* = 0.01) were significantly associated with hypertension (S2 Table). Being unemployed (aOR 0.3, 95% CI 0.1–0.9, *P* = 0.03), having monthly income 1–50 USD (aOR 0.2, 95% CI 0.1–0.5, *P* = 0.002), having monthly income 50–100 USD (aOR 0.2, 95% CI 0.1–0.7, *P* = 0.01 and being underweighted (aOR 0.3, 95% CI 0.9–3.2, *P* = 0.002) were significantly associated with lower risk of hypercholesterolemia (S3 Table).

## Discussion

To our knowledge, this is the first study in Cambodia to assess the prevalence and risk factors for non-communicable disease amongst people living with HIV. Several interesting findings should be noted. First, nearly half of the study population had a non-communicable disease, either as a single entity or in combination, with diabetes mellitus, hypertension and hypercholesterolemia increasing in prevalence in that order. The prevalence of diabetes mellitus, hypertension and hypercholesterolemia in people living with HIV was higher than that found in the general population for each of these disorders [21, 22]. Interestingly, the stepped increase in the proportions of participants with diabetes mellitus (at 8.8%), hypertension (at 15.1%) and hypercholesterolemia (at 34.7%) was also mirrored in the STEPS survey where the prevalence of diabetes mellitus, hypertension and hypercholesterolemia in the general population was 2.9%, 11.2% and 20.7%, respectively [22]. The findings from our study are in accordance with previous studies highlighting an excess of these diseases in people living with HIV who are initiated or not on ART [5, 8, 9].

Second, nearly 80% of these diseases were newly identified as a result of the study assessment, this being more common for patients with diabetes mellitus and hypercholesterolemia where a blood test was needed for diagnosis. These findings are not surprising as undiagnosed disease is also common in the general population. About half of the persons living with diabetes mellitus globally are undiagnosed [29], and surveys in Africa and Asia have found a high prevalence of undiagnosed hypertension [30, 31, 32]. Similarly, in the few studies that have prospectively measured blood pressure in people living with HIV, hypertension was newly diagnosed in 20–45% of cases [10, 11, 14].

Third, certain socio-demographic and behavioral factors such as living in an urban environment, working in an office and eating fewer vegetable servings put people living with HIV at risk of one or more non-communicable diseases. When assessing for each individual disease, some of these factors were uniformly significant, such as living in an urban environment, while others such as using lard for cooking were associated with one but not the other non-communicable disease. Urbanization is a recognized risk factor for non-communicable diseases worldwide [7, 33], so it is not surprising to see this as a uniform risk factor in our study. In previous studies, male gender, older age, higher BMI and increased abdominal fat have been significantly associated with a higher risk of hypertension [10, 11, 14]. We did not find these associations, although there was a non-significant trend between BMI and non-communicable diseases in people living with HIV. The proportions of participants in our survey who were overweight or obese were fairly similar to those found in the STEPS survey amongst the general population [22].

Finally, and surprisingly, a low level of physical exercise during leisure time appeared to be associated with a lower risk of non-communicable disease compared with moderate to high levels of exercise. We do not completely understand the reason, but it might relate to the question being contextually inappropriate or not precise enough to give a satisfactory response and /or the cross-sectional design of the study which makes it difficult to know whether the reporting of exercise levels preceded the NCD or vice versa. It is possible that people who engaged more in physical exercise were those who were diagnosed with an NCD and advised by their health providers to do regular exercise. People in rural areas may also be involved in significant amounts of physical activity that would protect them from conditions associated with a sedentary life style, yet they may not report that as exercise. This observation may also be related to potential recall and quantification bias.

The strengths of this study were the large sample size, the low rate of non-responders, full adherence to the study protocol, definition of terms and variables consistent with existing guidelines and previous studies and double-entry of data from the paper-based questionnaire into the electronic database software that minimized errors. The study was also conducted and reported in accordance with the Strengthening of Reporting of Observational studies in Epidemiology (STROBE) guidelines [34].

This study has some limitations. The cross-sectional design limits our understanding of causal links between risk factors and the development of non-communicable diseases, and in particular no distinctions can be made between determinants of new diseases and determinants of duration of diseases. The self-reported measures of behavior may lead to over- or under-reporting due to recall or social desirability bias as noted above. It is also likely that some of the measures used were not contextually appropriate. For example, low physical exercise during leisure time, which appeared to protect against non-communicable disease, may underestimate the amount of physical activity undertaken by people living in rural areas. The use of a portable capillary-based method of testing for blood glucose or cholesterol may have been less reliable than using laboratory-based methods, although the instrument used was regularly calibrated during the study period. We may also have underestimated the prevalence of diabetes mellitus by using fasting blood glucose measurements, as up to half the patients diagnosed with diabetes mellitus using the two-hour 75 Gram oral glucose tolerance in a large and recent study in China had normal fasting blood glucose levels [35]. Finally, the characteristics of our patient sample affected our analysis. For example, the fact that almost all of patients were on ART, and for at least 6 months, made it difficult to properly assess the effect of ART on non-communicable diseases. Finally, this was a cross-sectional study that assessed and identified elevated levels of blood glucose, blood pressure and cholesterol at one point in time. Sustained elevations of some of these factors, such as blood pressure, may be required to make a definitive diagnosis. Nevertheless, screening as employed in this study is an important first step in diagnosis of non-communicable diseases among people living with HIV in resource-poor settings.

Despite these limitations, findings from this study indicate that HIV programmes in Cambodia should consider introducing routine screening for non-communicable diseases for people living with HIV in care. The recent WHO guidelines recommend that ART should be offered to all people living with HIV regardless of clinical stage or CD4 cell count [36]. This means that eventually all people living with HIV will be in structured care with routine, regular follow-up. Introduction of screening for non-communicable diseases is therefore a feasible option. The concept of integrated care for patients with chronic diseases including HIV, diabetes mellitus and hypertension, has already been implemented in Cambodia [37], although in this instance, patients with their own diseases were managed separately within the same clinic. We suggest, however, that the screening, diagnosis and treatment of diabetes mellitus, hypertension, and hypercholesterolemia occur in the clinic for people living with HIV in care. This may impose too much of a workload on an already busy staff if implemented all at once, and a phased approach starting with blood pressure screening, may be targeted at first to persons at risk, and then moving to blood tests for biochemical measurements may be a way forward. Community health workers, who have been shown to perform simple clinical tasks while reducing the workload of facility-based health workers through task-shifting approaches [38], could also be utilized to perform screening. The implementation of these approaches, combined with high quality monitoring will be needed to learn lessons and inform policy and practice for future widespread scale up.

## Conclusions

This study found a high burden of non-communicable diseases among a sample of people living with HIV in Cambodia, with nearly half of the participants being found to have at least one non-communicable disease. The utility of active screening was evident in this study, where a majority of participants with these diseases were not aware of their conditions. As the non-communicable and HIV epidemics converge in Cambodia and other low- and middle-income counties, screening of non-communicable diseases should be integrated into routine HIV care, especially in urban areas, where co-morbidity risk might be higher compared to rural areas. Given the already existing high workload burden on health providers, deployment of community health workers to perform routine screening for non-communicable diseases among people living with HIV should be explored.

## Supporting information

S1 Table Associations between demographic, clinical and anthropometric characteristics and diabetes mellitus in 510 adults living with HIV in Cambodia in 2015. (DOCX)

S2 Table Associations between demographic, clinical and anthropometric characteristics and hypertension in 510 adults living with HIV in Cambodia in 2015. (DOCX)

S3 Table Associations between demographic, clinical and anthropometric characteristics and hypercholesterolemia in 510 adults living with HIV in Cambodia in 2015. (DOCX)

S1 Tool Questionnaire—English version. (DOCX)

S1 Data Minimal dataset. (DTA)
