# Supplementary material for: High prevalence of non-communicable diseases and associated risk factors amongst adults living with HIV in Cambodia
Source: PLoS One. 2017 Nov 9;12(11):e0187591. doi: 10.1371/journal.pone.0187591 (PMC5679628; doi:10.1371/journal.pone.0187591)
Supplement: S1 Table — (DOCX) [file pone.0187591.s001.docx]

**SUPPLEMENTARY INFORMATION**

S1 Table: Associations between demographic, clinical and anthropometric characteristics and diabetes mellitus in 510 adults living with HIV in Cambodia in 2015

| Characteristics at evaluation | | Total | Diabetes mellitus | | OR | (95% CI) | *P* value | aOR | (95% CI) | P value |
| --- | --- | --- | --- | --- | --- | --- | --- | --- | --- | --- |
|  |  | N | N | (%) |  |  |  |  |  |  |
| Age group in years: | |  |  |  |  |  |  |  |  |  |
|  | 22-30 | 14 | 2 | (14.3) | ref |  |  |  |  |  |
|  | 31-40 | 150 | 11 | (7.3) | 0.5 | (0.1-2.4) | 0.35 |  |  |  |
|  | 41-50 | 198 | 15 | (7.6) | 0.5 | (0.1-2.4) | 0.37 |  |  |  |
|  | 51 and Higher | 148 | 15 | (10.1) | 0.7 | (0.1-1.3) | 0.62 |  |  |  |
| Sex: |  |  |  |  |  |  |  |  |  |  |
|  | Male | 170 | 15 | (8.8) | ref |  |  |  |  |  |
|  | Female | 340 | 28 | (8.2) | 0.9 | (0.5-1.8) | 0.82 |  |  |  |
| Type of community: | |  |  |  |  |  |  |  |  |  |
|  | Rural | 202 | 17 | (8.4) | ref |  |  |  |  |  |
|  | Urban | 308 | 26 | (8.4) | 1.0 | (0.5-1.9) | 0.99 |  |  |  |
| Level of education: | |  |  |  |  |  |  |  |  |  |
|  | None | 115 | 15 | (13.0) | 1.9 | (1.0-3.8) | 0.04 | 1.9 | (0.9-4.1) | 0.05 |
|  | Schooling | 395 | 28 | (7.1) | ref |  |  |  |  |  |
| Occupation: | |  |  |  |  |  |  |  |  |  |
|  | Unemployed | 124 | 13 | (10.5) | 1.8 | (0.8-4.2) | 0.12 | 1.2 | (0.3-4.8) | 0.83 |
|  | Manual work | 221 | 13 | (5.9) | ref |  |  |  |  |  |
|  | Office work | 165 | 17 | (10.3) | 1.8 | (0.8-3.9) | 0.10 | 1.6 | (0.7-3.8) | 0.26 |
| Monthly income in past year ($): | | |  |  |  |  |  |  |  |  |
|  | None | 111 | 13 | (11.7) | ref |  |  |  |  |  |
|  | 1 – 50 | 170 | 10 | (5.9) | 0.5 | (0.2-1.1) | 0.08 | 0.3 | (0.1-1.5) | 0.18 |
|  | 51 – 100 | 110 | 9 | (8.2) | 0.6 | (0.2-1.6) | 0.38 | 0.4 | (0.1-1.8) | 0.23 |
|  | > 100 | 119 | 11 | (9.2) | 0.8 | (0.3-1.8) | 0.54 | 0.5 | (0.1-2.4) | 0.39 |
| Tobacco use: | |  |  |  |  |  |  |  |  |  |
|  | Never smoked | 377 | 30 | (8.0) | ref |  |  |  |  |  |
|  | Ex-smoker | 58 | 6 | (10.3) | 1.3 | (0.5-3.4) | 0.53 |  |  |  |
|  | Current smoker | 75 | 7 | (9.3) | 1.2 | (0.5-2.8) | 0.69 |  |  |  |
| Alcohol consumption: | |  |  |  |  |  |  |  |  |  |
|  | Never | 231 | 17 | (7.4) | ref |  |  |  |  |  |
|  | Ex-drinker | 66 | 7 | (10.6) | 1.5 | (0.6-3.8) | 0.39 |  |  |  |
|  | Current drinker | 213 | 19 | (8.9) | 1.2 | (0.6-2.4) | 0.54 |  |  |  |
| Fruit servings per day: | |  |  |  |  |  |  |  |  |  |
|  | None | 84 | 6 | (7.1) | 0.5 | (0.2-1.2) | 0.18 | 0.5 | (0.1-1.4) | 0.24 |
|  | 1 serving | 277 | 18 | (6.5) | 0.2 | (0.2-0.9) | 0.02 | 0.3 | (0.1-0.8) | 0.01 |
|  | 2 servings or more | 149 | 21 | (14.1) | ref |  |  |  |  |  |
| Vegetable servings per day: | |  |  |  |  |  |  |  |  |  |
|  | 1 serving | 57 | 5 | (8.8) | 1.0 | (0.4-2.8) | 0.92 |  |  |  |
|  | 2 serving or more | 453 | 38 | (8.4) | ref |  |  |  |  |  |
| Oil type used for cooking: | |  |  |  |  |  |  |  |  |  |
|  | None | 2 | 1 | (50.0) | 11.8 | (0.7-19.2) | 0.02 |  |  |  |
|  | Lard | 22 | 4 | (18.2) | 2.6 | (0.4-8.1) | 0.08 | 3.2 | (0.8-1.2) | 0.09 |
|  | Vegetable oil | 486 | 45 | (9.3) | ref |  |  |  |  |  |
| Physical activity in leisure time: | | |  |  |  |  |  |  |  |  |
|  | Low | 249 | 18 | (7.2) | 0.7 | (0.4-1.4) | 0.31 |  |  |  |
|  | Moderate | 25 | 2 | (8.0) | 0.8 | (0.2-3.6) | 0.77 |  |  |  |
|  | High | 236 | 23 | (9.7) | ref |  |  |  |  |  |
| Lifestyle advice from health- worker: | | |  |  |  |  |  |  |  |  |
|  | No | 263 | 19 | (7.2) | 1.4 | (0.7-2.6) | 0.31 |  |  |  |
|  | Yes | 247 | 24 | (9.7) | ref |  |  |  |  |  |
| Time since HIV diagnosis in months: | | |  |  |  |  |  |  |  |  |
|  | 12 – 24 | 17 | 1 | (5.9) | ref |  |  |  |  |  |
|  | 25 and above | 493 | 42 | (8.5) | 1.5 | (0.2-11.5) | 0.70 |  |  |  |
| ART status : | |  |  |  |  |  |  |  |  |  |
|  | Not on ART | 17 | 2 | (11.8) | ref |  |  |  |  |  |
|  | On ART | 493 | 41 | (8.3) | 0.7 | (0.2-3.0) | 0.61 |  |  |  |
| Length on ART in months (n=493): | | |  |  |  |  |  |  |  |  |
|  | 6 – 12 | 14 | 0 | (0.0) | ref |  |  |  |  |  |
|  | 13 – 60 | 121 | 10 | (8.3) | inf | inf |  |  |  |  |
|  | 61 and above | 358 | 31 | (8.7) | inf | inf | 0.66 |  |  |  |
| Type of ART Regimen (n=493): | | |  |  |  |  |  |  |  |  |
|  | ART with PI | 35 | 2 | (5.7) | 0.6 | (0.1-2.8) | 0.56 |  |  |  |
|  | ART without PI | 458 | 39 | (8.5) | ref |  |  |  |  |  |
| Weight (BMI) at evaluation: | |  |  |  |  |  |  |  |  |  |
|  | Underweight | 99 | 3 | (3.0) | 0.3 | (0.2-1.2) | 0.07 | 0.3 | (0.1-1.0) | 0.07 |
|  | Normal | 302 | 25 | (8.3) | ref |  |  |  |  |  |
|  | Overweight | 88 | 10 | (11.4) | 1.4 | (0.6-3.1) | 0.37 | 1.6 | (0.6-4.1) | 0.34 |
|  | Obese | 21 | 5 | (23.8) | 3.5 | (1.2-10.2) | 0.01 | 3.6 | (0.9-13.3) | 0.06 |
| Abdominal obesity | |  |  |  |  |  |  |  |  |  |
|  | Obese | 99 | 13 | (13.1) | 1.9 | (0.9-3.8) | 0.06 | 1.0 | (0.4-2.4) | 0.91 |
|  | Non-obese | 411 | 30 | (7.3) | ref |  |  |  |  |  |

OR = odds ratio; CI = confidence interval; aOR = adjusted odds ratio; ART = antiretroviral therapy; PI = protease inhibitor; BMI = body mass index
