# Supplementary material for: High prevalence of non-communicable diseases and associated risk factors amongst adults living with HIV in Cambodia
Source: PLoS One. 2017 Nov 9;12(11):e0187591. doi: 10.1371/journal.pone.0187591 (PMC5679628; doi:10.1371/journal.pone.0187591)
Supplement: S2 Table — (DOCX) [file pone.0187591.s002.docx]

**SUPPLEMENTARY INFORMATION**

**S2 Table: Associations between demographic, clinical and anthropometric characteristics and hypertension in 510 adults living with HIV in Cambodia in 2015**

| Characteristics at evaluation | | Total | Hypertension | | OR | (95% CI) | *P* value | aOR | (95% CI) | P value |
| --- | --- | --- | --- | --- | --- | --- | --- | --- | --- | --- |
|  |  | n | N | (%) |  |  |  |  |  |  |
| Age group in years: | |  |  |  |  |  |  |  |  |  |
|  | 22-30 | 14 | 1 | (7.1) | ref |  |  |  |  |  |
|  | 31-40 | 150 | 19 | (10.7) | 1.5 | (0.2-12.6) | 0.67 | 1.9 | (0.2-17.7) | 0.55 |
|  | 41-50 | 198 | 38 | (19.2) | 3.1 | (0.4-24.3) | 0.26 | 2.5 | (0.2-22.8) | 0.39 |
|  | 51 and Higher | 148 | 22 | (14.9) | 2.3 | (0.3-18.2) | 0.42 | 2.4 | (0.2-22.3) | 0.41 |
| Sex: |  |  |  |  |  |  |  |  |  |  |
|  | Male | 170 | 27 | (15.9) | ref |  |  |  |  |  |
|  | Female | 340 | 50 | (14.7) | 0.9 | (0.5-1.5) | 0.72 |  |  |  |
| Type of community: | |  |  |  |  |  |  |  |  |  |
|  | Rural | 202 | 3 | (1.5) | ref |  |  |  |  |  |
|  | Urban | 308 | 74 | (24.0) | 21.0 | (6.5-67.6) | <0.001 | 19.0 | (5.5-65.0) | <0.001 |
| Level of education: | |  |  |  |  |  |  |  |  |  |
|  | None | 115 | 14 | (12.2) | 0.7 | (0.4-1.7) | 0.31 |  |  |  |
|  | Schooling | 395 | 63 | (15.9) | ref |  |  |  |  |  |
| Occupation: | |  |  |  |  |  |  |  |  |  |
|  | Unemployed | 124 | 18 | (14.5) | 1.1 | (0.6-2.0) | 0.80 |  |  |  |
|  | Manual work | 221 | 30 | (13.6) | ref |  |  |  |  |  |
|  | Office work | 165 | 29 | (17.6) | 1.4 | (0.8-2.4) | 0.27 |  |  |  |
| Monthly income in past year ($): | | |  |  |  |  |  |  |  |  |
|  | None | 111 | 12 | (10.8) | ref |  |  |  |  |  |
|  | 1 – 50 | 170 | 23 | (13.5) | 1.3 | (0.6-2.7) | 0.49 | 1.5 | (0.7-3.5) | 0.36 |
|  | 51 – 100 | 110 | 19 | (17.3) | 1.7 | (0.8-3.7) | 0.16 | 1.8 | (0.7-4.3) | 0.31 |
|  | > 100 | 119 | 23 | (19.3) | 2.0 | (0.9-4.2) | 0.1 | 1.5 | (0.7-3.5) | 0.46 |
| Tobacco use: | |  |  |  |  |  |  |  |  |  |
|  | Never smoked | 377 | 54 | (14.3) | ref |  |  |  |  |  |
|  | Ex-smoker | 58 | 12 | (20.7) | 1.6 | (0.8-3.1) | 0.07 |  |  |  |
|  | Current smoker | 75 | 11 | (14.7) | 1.0 | (0.5-2.1) | 0.20 |  |  |  |
| Alcohol consumption: | |  |  |  |  |  |  |  |  |  |
|  | Never | 231 | 34 | (14.7) | ref |  |  |  |  |  |
|  | Ex-drinker | 66 | 10 | (15.2) | 1.0 | (0.5-2.2) | 0.93 |  |  |  |
|  | Current drinker | 213 | 33 | (15.5) | 1.1 | (0.6-1.8) | 0.81 |  |  |  |
| Fruit servings per day: | |  |  |  |  |  |  |  |  |  |
|  | None | 84 | 10 | (11.9) | 0.5 | (0.2-1.0) | 0.05 | 0.5 | (0.2-1.2) | 0.07 |
|  | 1 serving | 277 | 34 | (12.3) | 0.5 | (0.3-0.8) | 0.007 | 0.5 | (0.3-1.0) | 0.02 |
|  | 2 servings or more | 149 | 33 | (22.1) | ref |  |  |  |  |  |
| Vegetable servings per day: | |  |  |  |  |  |  |  |  |  |
|  | 1 serving | 57 | 16 | (28.1) | 2.5 | (1.3-4.7) | 0.003 | 2.0 | (0.9-4.4) | 0.07 |
|  | 2 serving or more | 453 | 61 | (13.5) | ref |  |  |  |  |  |
| Oil type used for cooking: | |  |  |  |  |  |  |  |  |  |
|  | None | 2 | 1 | (50.0) | 6.0 | (0.4-97.7) | 0.14 | 3.6 | (0.1- 93.1) | 0.41 |
|  | Lard | 22 | 7 | (31.8) | 2.8 | (1.1-7.1) | 0.02 | 4.8 | (1.4-16.5) | 0.01 |
|  | Vegetable oil | 486 | 69 | (14.2) | ref |  |  |  |  |  |
| Physical activity in leisure time: | | |  |  |  |  |  |  |  |  |
|  | Low | 249 | 17 | (6.8) | 0.2 | (0.14-0.4) | <0.001 | 0.3 | (0.2-0.7) | 0.007 |
|  | Moderate | 25 | 5 | (20.0) | 0.8 | (0.3-2.3) | 0.70 | 0.9 | (0.3-2.9) | 0.97 |
|  | High | 236 | 55 | (23.3) | ref |  |  |  |  |  |
| Lifestyle advice from health- worker: | | |  |  |  |  |  |  |  |  |
|  | No | 263 | 33 | (12.5) | 0.7 | (0.4-1.1) | 0.09 | 0.9 | (0.5-1.6) | 0.64 |
|  | Yes | 247 | 44 | (17.8) | ref |  |  |  |  |  |
| Time since HIV diagnosis in months: | | |  |  |  |  |  |  |  |  |
|  | 12 – 24 | 17 | 1 | (5.9) | ref |  |  |  |  |  |
|  | 25 and above | 493 | 76 | (15.4) | 2.9 | (0.4-22.3) | 0.28 |  |  |  |
| ART status: | |  |  |  |  |  |  |  |  |  |
|  | Not on ART | 17 | 1 | (5.9) | ref |  |  |  |  |  |
|  | On ART | 493 | 76 | (15.4) | 2.9 | (0.4-22.3) | 0.28 |  |  |  |
| Length on ART in months (n=493): | | | |  |  |  |  |  |  |  |
|  | 6 – 12 | 14 | 4 | (30.8) | ref |  |  |  |  |  |
|  | 13 – 60 | 121 | 19 | (15.7) | 0.4 | (0.1-1.5) | 0.19 | 0.4 | (0.1-1.6) | 0.47 |
|  | 61 and above | 358 | 53 | (14.8) | 0.4 | (0.1-1.3) | 0.16 | 0.4 | (0.1-1.5) | 0.49 |
| Type of ART Regimen (n=493): | | |  |  |  |  |  |  |  |  |
|  | ART with PI | 35 | 5 | (14.3) | 0.9 | (0.3-2.4) | 0.84 |  |  |  |
|  | ART without PI | 458 | 71 | (15.5) | ref |  |  |  |  |  |
| Weight (BMI) at evaluation: | | 510 |  |  |  |  |  |  |  |  |
|  | Underweight | 99 | 12 | (12.1) | 0.8 | (0.4-1.6) | 0.54 |  |  |  |
|  | Normal | 302 | 44 | (14.6) | ref |  |  |  |  |  |
|  | Overweight | 88 | 16 | (18.2) | 1.3 | (0.7-2.4) | 0.40 |  |  |  |
|  | Obese | 21 | 5 | (23.8) | 1.8 | (0.6-5.3) | 0.25 |  |  |  |
| Abdominal obesity | | 510 |  |  |  |  |  |  |  |  |
|  | Obese | 99 | 17 | (17.2) | 1.2 | (0.7-7.2) | 0.52 |  |  |  |
|  | Non-obese | 411 | 60 | (14.5) | ref |  |  |  |  |  |

OR = odds ratio; CI = confidence interval; aOR = adjusted odds ratio; ART = antiretroviral therapy; PI = protease inhibitor; BMI = body mass index
