# Supplementary material for: High prevalence of non-communicable diseases and associated risk factors amongst adults living with HIV in Cambodia
Source: PLoS One. 2017 Nov 9;12(11):e0187591. doi: 10.1371/journal.pone.0187591 (PMC5679628; doi:10.1371/journal.pone.0187591)
Supplement: S3 Table — (DOCX) [file pone.0187591.s003.docx]

**SUPPLEMENTARY INFORMATION**

S3 Table: Associations between demographic, clinical and anthropometric characteristics and hypercholesterolemia in 510 adults living with HIV in Cambodia in 2015

| Characteristics at evaluation | | Total | Hypercholesterolemia | | OR | (95% CI) | P value | AOR | (95% CI) | P value |
| --- | --- | --- | --- | --- | --- | --- | --- | --- | --- | --- |
|  |  | n | n | (%) |  |  |  |  |  |  |
| Age group in years: | |  |  |  |  |  |  |  |  |  |
|  | 22-30 | 14 | 4 | (28.6) | ref |  |  |  |  |  |
|  | 31-40 | 150 | 42 | (28.0) | 0.9 | (0.3-3.3) | 0.96 | 0.9 | (0.2-3.5) | 0.95 |
|  | 41-50 | 198 | 65 | (32.8) | 1.8 | (0.4-4.0) | 0.74 | 1.3 | (0.3-4.9) | 0.64 |
|  | 51 and Higher | 148 | 66 | (44.6) | 2.0 | (0.6-6.7) | 0.24 | 2.3 | (0.6-8.7) | 0.19 |
| Sex: |  |  |  |  |  |  |  |  |  |  |
|  | Male | 170 | 69 | (40.6) | ref |  |  |  |  |  |
|  | Female | 340 | 108 | (31.8) | 0.7 | (0.5-1.0) | 0.04 | 0.9 | (0.5-1.7) | 0.89 |
| Type of community: | |  |  |  |  |  |  |  |  |  |
|  | Rural | 202 | 62 | (30.7) | ref |  |  |  |  |  |
|  | Urban | 308 | 115 | (37.3) | 1.3 | (0.9-1.3) | 0.04 | 1.0 | (0.6-1.7) | 0.73 |
| Level of education: | |  |  |  |  |  |  |  |  |  |
|  | None | 115 | 36 | (31.3) | 0.8 | (0.5-1.3) | 0.12 |  |  |  |
|  | Schooling | 395 | 141 | (35.5) | Ref |  |  |  |  |  |
| Occupation: | |  |  |  |  |  |  |  |  |  |
|  | Unemployed | 124 | 40 | (32.3) | 1.0 | (0.6-1.6) | 0.98 | 0.3 | (0.1-0.9) | 0.03 |
|  | Manual work | 221 | 71 | (32.1) | ref |  |  |  |  |  |
|  | Office work | 165 | 66 | (40.0) | 0.7 | (0.5-1.0) | 0.10 | 1.1 | (0.7-1.8) | 0.53 |
| Monthly income in past year ($): | | |  |  |  |  |  |  |  |  |
|  | None | 111 | 42 | (37.8) | ref |  |  |  |  |  |
|  | 1 – 50 | 170 | 46 | (27.1) | 0.6 | (0.4-1.0) | 0.05 | 0.2 | (0.1-0.5) | 0.002 |
|  | 51 – 100 | 110 | 37 | (33.6) | 1.2 | (0.7-2.0) | 0.51 | 0.2 | (0.1-0.7) | 0.01 |
|  | > 100 | 119 | 52 | (43.7) | 1.2 | (0.7-2.1) | 0.36 | 0.4 | (0.1-1.0) | 0.07 |
| Tobacco use: | |  |  |  |  |  |  |  |  |  |
|  | Never smoked | 377 | 125 | (33.2) | ref |  |  |  |  |  |
|  | Ex-smoker | 58 | 28 | (48.3) | 1.8 | (1.0-3.3) | 0.02 | 1.4 | (0.7-3.0) | 0.29 |
|  | Current smoker | 75 | 24 | (32.0) | 0.9 | (0.6-1.6) | 0.84 | 0.8 | (0.4-1.8) | 0.72 |
| Alcohol consumption: | |  |  |  |  |  |  |  |  |  |
|  | Never | 231 | 73 | (31.6) | ref |  |  |  |  |  |
|  | Ex-drinker | 66 | 20 | (30.3) | 0.9 | (0.5-1.7) | 0.84 | 0.9 | (0.5-1.8) | 0.94 |
|  | Current drinker | 213 | 84 | (39.4) | 0.7 | (0.5-1.0) | 0.08 | 1.5 | (0.9-2.4) | 0.08 |
| Fruit servings per day: | |  |  |  |  |  |  |  |  |  |
|  | None | 84 | 25 | (29.8) | 0.8 | (0.5-1.4) | 0.48 |  |  |  |
|  | 1 serving | 277 | 101 | (36.5) | 1.1 | (0.7-1.7) | 0.64 |  |  |  |
|  | 2 servings or more | 149 | 51 | (34.2) | ref |  |  |  |  |  |
| Vegetable servings per day: | |  |  |  |  |  |  |  |  |  |
|  | 1 serving | 57 | 28 | (49.1) | 1.9 | (1.1-3.4 | 0.01 | 1.7 | (0.9-3.1) | 0.09 |
|  | 2 serving or more | 453 | 149 | (32.9) | ref |  |  |  |  |  |
| Oil type used for cooking: | |  |  |  |  |  |  |  |  |  |
|  | None | 2 | 2 | (100.0) | - | - | - |  |  |  |
|  | Lard | 22 | 10 | (45.5) | 1.6 | (0.7-3.8) | 0.26 | 1.5 | (0.5-3.9) | 0.40 |
|  | Vegetable oil | 486 | 165 | (34.0) | ref |  |  |  |  |  |
| Physical activity in leisure time: | | |  |  |  |  |  |  |  |  |
|  | Low | 249 | 78 | (31.3) | 0.8 | (0.5-1.2) | 0.27 | 0.7 | (0.5-1.2) | 0.31 |
|  | Moderate | 25 | 14 | (56.0) | 2.2 | (0.8-5.2) | 0.05 | 2.2 | (0.8-5.5) | 0.09 |
|  | High | 236 | 85 | (36.0) | ref |  |  |  |  |  |
| Lifestyle advice from health- worker: | | |  |  |  |  |  |  |  |  |
|  | No | 263 | 90 | (34.2) | 0.9 | (0.6-1.4) | 0.81 |  |  |  |
|  | Yes | 247 | 87 | (35.2) | ref |  |  |  |  |  |
| Time since HIV diagnosis in months: | | |  |  |  |  |  |  |  |  |
|  | 12 – 24 | 17 | 4 | (23.5) | ref |  |  |  |  |  |
|  | 25 and above | 493 | 173 | (35.1) | 1.7 | (0.5-5.5) | 0.32 |  |  |  |
| ART status: | |  |  |  |  |  |  |  |  |  |
|  | Not on ART | 17 | 5 | (29.4) | ref |  |  |  |  |  |
|  | On ART | 493 | 172 | (34.9) | 1.3 | (0.4-3.7) | 0.64 |  |  |  |
| Length on ART in months (n=493): | | |  |  |  |  |  |  |  |  |
|  | 6 – 12 | 14 | 0 | (0.0) | ref |  |  |  |  |  |
|  | 13 – 60 | 121 | 38 | (31.4) | inf | inf | 0.34 |  |  |  |
|  | 61 and above | 358 | 133 | (37.2) | inf | inf | 0.27 |  |  |  |
| Type of ART Regimen (n=493): | | |  |  |  |  |  |  |  |  |
|  | ART with PI | 35 | 15 | (42.9) | 1.4 | (0.7-2.8) | 0.30 |  |  |  |
|  | ART without PI | 458 | 157 | (34.3) | ref |  |  |  |  |  |
| Weight (BMI) at evaluation: | |  |  |  |  |  |  |  |  |  |
|  | Underweight | 99 | 18 | (18.2) | 0.4 | (0.2-0.7) | 0.001 | 0.3 | (0.2-0.6) | 0.002 |
|  | Normal | 302 | 106 | (15.1) | ref |  |  |  |  |  |
|  | Overweight | 88 | 41 | (46.5) | 1.6 | (1.0-2.6) | 0.05 | 1.7 | (0.9-3.2) | 0.05 |
|  | Obese | 21 | 12 | (57.1) | 2.5 | (1.0-6.0) | 0.04 | 2.9 | (1.0-8.1) | 0.03 |
| Abdominal obesity | |  |  |  |  |  |  |  |  |  |
|  | Obese | 99 | 41 | (41.5) | 1.4 | (0.9-2.2) | 0.11 | 0.8 | (0.4-1.5) | 0.52 |
|  | Non-obese | 411 | 136 | (33.1) | ref |  |  |  |  |  |

OR = odds ratio; CI = confidence interval; aOR = adjusted odds ratio; ART = antiretroviral therapy; PI = protease inhibitor; BMI = body mass index
