# Supplementary material for: High prevalence of non-communicable diseases and associated risk factors amongst adults living with HIV in Cambodia
Source: PLoS One. 2017 Nov 9;12(11):e0187591. doi: 10.1371/journal.pone.0187591 (PMC5679628; doi:10.1371/journal.pone.0187591)
Supplement: S1 Tool — (DOCX) [file pone.0187591.s004.docx]

**S1 Tool**

**Behavioral Questionnaire**

For more information please contact Dr. Yi Siyan Research Director of KHANA organization

Mobile Phone: (855) 012 417 170

Tel: (855) 23 211 505

Fax: (855) 023 214 049

Address: #33, Street 71 Phnom Penh, Cambodia Box 2311 Phnom Penh 3

Email: [ysiyan@khana.org.kh](mailto:ysiyan@khana.org.kh)

**Survey Information**

| Provincial Code | **□□**  Provincial Coding:  Phnom Penh 01  Takeo 02  Pursat 03  Battambang 04  Siem Reap 05 |
| --- | --- |
| Community Type: | 1. Rural 2. Semi-urban 3. Urban |
| Health Center (code) | **□□** |
| Date of Interview | DD/MM/YY: ………/ …………/ …………. |
| Interviewer Code | **□□** |
| Respondent Group Code | 1. On ART 2. On Pre-ART |
| Field Supervisor code |  |
| Data entry clerk 1 |  |
| Data entry clerk 2 |  |
| Checker/ Cleaner/ Date | ………………………………………………………  DD/MM/YY: ………/ …………/ …………. |

**Demographics Information (DI7):**

| Code | Questions | Response |
| --- | --- | --- |
| DI01 | Residential Address | Commune: …………………………..  District: ………………………………  Province: ……………………………. |
| DI 02 | Sex | 1. Male 2. Female 3. Other_________________ |
| DI 03 | Age | ………… years |
| DI 04 | Marital status | 1. Single 2. Married 3. Divorced 4. Widowed 5. Separated 6. Cohabiting 7. Other(specify)________________ |
| DI 05 | What is the highest level of education attained? | 1. No schooling 2. Completed Primary 3. Completed Secondary 4. Completed high school 5. Completed undergraduate 6. Completed post graduate |
| DI 06 | What is your main occupation? | 1. Unemployed 2. Student 3. Moto/driver 4. Farmer 5. Worker 6. Own business 7. Junior officer(GOs, Private, NGO) 8. Junior protector(Police, soldier) 9. Entertainment worker 10. Other______________________ |
| DI 07 | What is your monthly income in the last 12mont? (USD) | ………………… USD |

**Behavioral Measurements:**

**Tobacco Use**

| Code | Questions | Response |
| --- | --- | --- |
| BM 01 | Do you currently smoke or use any tobacco products such as cigarettes, cigars, pipes, snuff, and chew tobacco? | 1. Yes 2. No (go to BM05) |
| BM 02 | Do you currently smoke tobacco products daily? | 1. Yes 2. No |
| BM 03 | How old were you when you first started smoking or using tobacco regularly? | _________years  Code 99 if doesn't remember |
| BM 04 | On average, how many of the following do you smoke each day? | 1. Manufactured cigarettes _____ 2. Hand rolled cigarettes _____ 3. Pipe fillings of tobacco _____ (when finish go to BM 07) |
| BM 05 | If not a current smoker, did you ever smoke regularly? | 1. Yes 2. No **(go to BM 07)** |
| BM 06 | If yes, how old were you when you stopped | _________year  Code 99 if doesn't remember |
| BM 07 | Do you currently use any smokeless tobacco such as snuff or chewing tobacco? | 1. Yes 2. No **(go to BM 09)** |
| BM 08 | If yes, on average how many times a day do you use…  (record for each type) | 1. Snuff, by mouth ____Times 2. Snuff, by nose ____ Times 3. Chewing tobacco ____ Times |

**Alcohol Consumption**

| Code | Questions | Response |
| --- | --- | --- |
| BM 09 | Have you ever consumed a drink that contains alcohol such as beer, wine, spirit, fermented cider, etc.? | 1. Yes 2. No (Go to BM 13) |
| BM 10 | Have you consumed alcohol within the past 12 months? | 1. Yes 2. No (Go to BM 13) |
| BM 11 | In the past 12 months, how frequently have you had at least one drink? | 1. 5 or more days per week 2. 1-4 days/week 3. 1-3 days/month 4. Less than once a month |
| BM 12 | During each day of the past 7 days, how many standard drinks of any alcohol did you have each day? | 1. Monday __________ 2. Tuesday __________ 3. Wednesday __________ 4. Thursday __________ 5. Friday __________ 6. Saturday __________ 7. Sunday __________   Code 99 for don’t know. |

**Diet**

| Code | Questions | Response |
| --- | --- | --- |
| BM 13 | In a typical week, how many days do you eat fruit? | Number of days _____ (If don’t know code: 77)  (If 0 skip to BM 15) |
| BM 14 | On a typical day that you eat fruits, how many servings of fruits do you have? | Number of servings _____  (If don’t know code: 77) |
| BM 15 | In a typical week, on how many days do you eat vegetables? | Number of days _____  (If don’t know code: 77) |
| BM 16 | On a typically day that you eat vegetables, how many servings of vegetables do you have? | Number of servings _____  (If don’t know code: 77) |
| BM 17 | Are your meals usually prepared at home? | 1. Yes 2. No |
| BM 18 | What types of oils or fat is most often used for meal preparation in your household? (Only one answer) | 1. Vegetable oil 2. Lard 3. Butter 4. Margarine 5. Peanut oil 6. Fish oil 7. None in particular 8. None used 9. Other, specify: _____________ |

**Physical Activity**

| Code | Questions | Response |
| --- | --- | --- |
| BM 19 | Does your work involve mostly sitting or standing with walking for no more than 10 minutes at a time? | 1. Yes 2. No |
| BM 20 | Does your work involve vigorous activity like heavy lifting, digging or construction work for at least 10 minutes at a time? | 1. Yes 2. No (Go to BM 23) |
| BM 21 | In a typical week, on how many days do you do vigorous activities as part of your work? | Days a week: _____ |
| BM 22 | On a typical day on which you do vigorous activity, how much time do you spend doing such work? | In hours ______Minute______ |
| BM 23 | How long is your typical working day? | Number of hours _____Minute____ |
| Other than activities that you have already mentioned, I would like to ask you about the way you travel to and from places. For example to work, for shopping, to the field, to market, to church, funerals, gatherings. | | |
| BM 24 | Do you walk or use a bicycle (pedal cycle) for at least 10 minutes continuously to get to and from places? | 1. Yes 2. No |
| BM 25 | In your leisure time, do you do any vigorous activities like running or weight lifting strenuous sports for at least 10 minutes at a time? | 1. Yes 2. No (Go to Q BM 28) |
| BM 26 | If yes, in a typical week, on how many days do you do vigorous activities as part of your leisure time. | Days a week _____ |
| BM 27 | How much time do you spend doing this (vigorous activities) on a typical day? | In hours and minutes:  ________ _________ |
| BM 28 | In your leisure time, do you do any moderate activities such as brisk walking, cycling for at least 10 minutes at a time? | 1. Yes 2. No (Go to Q BM 31) |
| BM 29 | If yes, in a typical week, on how many days do you do moderate activities as part of leisure time? | Days a week ________ |

| BM 30 | How much time do you spend doing this (moderate activities) on a typical day? | In hours and minutes:  ________ _________ |
| --- | --- | --- |
| The following question is about sitting or reclining. Think back over the past 7 days, to the time spent at work, at home, in leisure, including time spent sitting at a desk, visiting friends, reading, or watching television, but do not include time spent sleeping. | | |
| BM 31 | Over the past 7 days, how much time did you spend sitting or reclining on a typical day? | In hours and minutes:  ________ _________ |
| **EXPANDED: HISTORY OF HIGH BLOOD PRESSURE**  Now we are going to ask you questions about your history of blood pressure. | | |
| BM 32 | When was your blood pressure last measured by a health professional? | 1. Within past 12 months 2. 1-5 years ago 3. Over past 5 years 4. Never had my blood pressure checked before |
| BM 33 | During the past 12 months have you been told by a doctor or other health worker that you have elevated blood pressure or hypertension? | 1. Yes 2. No (Go to Q BM41) |
| Now we are going to ask you questions about treatments for high blood pressure prescribed by a doctor or other health worker. | | |
| BM 34 | Have you been taking any drugs prescribed by a doctor or health worker in the past 2 weeks? | 1. Yes 2. No |
| BM 35 | Have you been on any special prescribed diet including salt reduction? | 1. Yes 2. No |
| BM 36 | Were you given advice or treatment to lose weight? | 1. Yes 2. No |
| BM 37 | Were you given advice to stop smoking? | 1. Yes 2. No |
| BM 38 | Were you given advice to start or do more exercise? | 1. Yes 2. No |
| BM 39 | During the past 12 months have you consulted any traditional healer for elevated blood pressure or hypertension? | 1. Yes 2. No |
| BM 40 | Are you currently taking any herbal or traditional remedy for your high blood pressure? | 1. Yes 2. No |
| BM 41 | Have your family had elevated blood pressure or hypertension? | 1. Yes 2. No |
| **EXPANDED: HISTORY OF DIABETES**  Now we are going to ask you questions about your history of diabetes. | | |
| BM 42 | Have you had your blood sugar measured in the past 12 months? | 1. Yes 2. No |
| BM 43 | Have you ever been diagnosed by a doctor or other health worker that you have diabetes? | 1. Yes 2. No (Skip to BM 53) |
| BM 44 | Are you currently receiving any of the following treatments for diabetes prescribed by a doctor or other health worker: | |
| BM 45 | Are you on Insulin injections? | 1. Yes 2. No |
| BM 46 | Are you on any oral drugs that you have taken in the past 2 weeks? | 1. Yes 2. No |
| BM 47 | Are you on any special prescribed diet? | 1. Yes 2. No |
| BM 48 | Have you been given advice or treatment to lose weight? | 1. Yes 2. No |
| BM 49 | Have you been advised to stop smoking? | 1. Yes 2. No |
| BM 50 | Have you been given advice to start or do more exercise? | 1. Yes 2. No |
| BM 51 | During the past 12 months have you consulted a traditional healer/faith healer for diabetes? | 1. Yes 2. No |
| BM 52 | Are you currently taking any herbal or traditional remedy for your diabetes? | 1. Yes 2. No |
| BM 53 | Have your family had diabetes? | 1. Yes 2. No |
| **History of Raised Total Cholesterol** | | |
| BM 54 | Have you ever had your cholesterol (fat levels in your blood) measured by a doctor or other health worker? | 1. Yes 2. No |
| BM 55 | Have you ever been told by a doctor or other health worker that you have raised cholesterol? | 1. Yes 2. No |
| BM 56 | Have you been told in the past 12 months? | 1. Yes 2. No (Skip to BM 60) |
| BM 57 | In the past two weeks, have you taken any oral treatment (medication) for raised total cholesterol prescribed by a doctor or other health worker? | 1. Yes 2. No |
| BM 58 | Have you ever seen a traditional healer for raised cholesterol? | 1. Yes 2. No |
| BM 59 | Are you currently taking any herbal or traditional remedy for your raised cholesterol? | 1. Yes 2. No |
| **Lifestyle Advice** | | |
| *BM 60* | **In the past 3 years leave you been told by doctor or other health worker to:** | |
| *BM 60.1* | Quit using tobacco or don’t start | 1. Yes 2. No |
| *BM 60.2* | Reduce salt in your diet | 1. Yes 2. No |
| *BM 60.3* | Eat at least five servings of fruit and/or vegetables each day | 1. Yes 2. No |
| *BM 60.4* | Reduce fat in your diet | 1. Yes 2. No |
| *BM 60.5* | Start or do more physical activity | 1. Yes 2. No |
| *BM 60.6* | Maintain a healthy body weight or lose weight | 1. Yes 2. No |

**HIV and ART Inventory**

| Code | Questions | Response (SKIP) |
| --- | --- | --- |
| BM 61 | How long have you been living with HIV? | Months_________ |
| BM 62 | Are you currently on ART? | 1. Yes 2. No (Skip to BM 64) |
| BM 63 | How long have you been on antiretroviral treatment? | Months_________ |
| BM 64 | Are you currently on any medication to prevent or to treat opportunistic infections? | 1. Yes 2. No |
| BM 65 | Are you currently taking tuberculosis treatment? | 1. Yes 2. No |
| BM 66 | During the past 6 months, have you received blood test for CD4 count? | 1. Yes 2. No (Skip to BM 68) 3. Don’t know (Skip to BM 68) |
| BM 67 | What was your CD4 count when you received the most recent blood test? | CD4 count _________ (00 if do not know) |

**THIS SECTION IS TO BE COMPLETED BY THE PATIENT AND STUDY PERSONNEL TOGETHER.**

**BM 68**.You are currently taking the following drugs at the frequency and doses listed.

| Study Drug Name/Dose | # Pills Each Time  (Pills Each Dose) | # Times Per Day  (Doses Per Day) |
| --- | --- | --- |
|  |  |  |
|  |  |  |
|  |  |  |
|  |  |  |
|  |  |  |
|  |  |  |
|  |  |  |
|  |  |  |
|  |  |  |
|  |  |  |
|  |  |  |
|  |  |  |
|  |  |  |
|  |  |  |
|  |  |  |

The next section of the questionnaire asks about your HIV study medications that you took over the last four days.

**BM 69**. The next section of the questionnaire asks about the study medications that you may have missed taking over the last four days. Please complete the following table by filling in the boxes below.

IF YOU TOOK ONLY A PORTION OF A DOSE ON ONE OR MORE OF THESE DAYS, PLEASE REPORT THE DOSE(S) AS BEING MISSED.

| Step 1  Names of your anti-HIV study drugs | HOW MANY DOSES DID YOU MISS… | | | |
| --- | --- | --- | --- | --- |
|  | Step 2  Yesterday | Step 3  Day before yesterday  (2 days ago) | Step 4  3 days ago | Step 5  4 days ago |
|  | □ Doses | □ Doses | □ Doses | □ Doses |
|  | □ Doses | □ Doses | □ Doses | □ Doses |
|  | □ Doses | □ Doses | □ Doses | □ Doses |
|  | □ Doses | □ Doses | □ Doses | □ Doses |
|  | □ Doses | □ Doses | □ Doses | □ Doses |
|  | □ Doses | □ Doses | □ Doses | □ Doses |
|  | □ Doses | □ Doses | □ Doses | □ Doses |

The following questions pertain to the study regimen on page 2.

If you took only a portion of a dose on one or more of these days, please report the dose(s) as being missed.

**BM 70**. During the past 4 days, on how many days have you missed taking all your doses?

None One Day Two Days Three Days Four Days

**BM 71**. Most anti-HIV medication need to be taken on a schedule, such as “2 times a day”or “3 times a day” or “every 8 hours.” How closely did you follow your specific schedule over the last four days?

Never Some of about half Most of All of

the time of the time the time the time

□ 0 □ 1 □ 2 □ 3 □ 4

**BM 72**.Do any of your anti-HIV medications have special instructions, such as “take with food” or “on an empty stomach” or “with plenty of fluids?”

□ 1 Yes □ 2 No (Skip to BM 74)

**BM** 73.If yes how often did you follow this instruction over the last four day

Never Some of about half Most of All of

the time of the time the time the time

□ 0 □ 1 □ 2 □ 3 □ 4

**BM 74**. Some people find that they forget to take their pills on the weekend days. Did you miss any of your anti-HIV medications last weekend – last Saturday or Sunday?

□ 1 Yes □ 2 No

**BM 75**. When was the last time you missed any of your medications? Check one.

□ 5 Within the past week

□ 4 1-2 weeks ago

□ 3 2-4 weeks ago

□ 2 1-3 months ago

□ 1 More than 3 months ago

□ 0 Never skip medications or not applicable

**Anthropometric and Biochemical Questionnaire**

For more information please contact Dr. Yi Siyan Research Director of KHANA organization

Mobile Phone: (855) 012 417 170

Tel: (855) 23 211 505

Fax: (855) 023 214 049

Address: #33, Street 71 Phnom Penh, Cambodia Box 2311 Phnom Penh 3

Email: ysiyan@khana.org.kh

**Survey Information**

| ID |  |
| --- | --- |
| Date of Interview | DD/MM/YY: ………/ …………/ …………. |
| Hospital Code | ____/____ |
| Data entry clerk 1 |  |
| Data entry clerk 2 |  |
| Checker/ Cleaner/ Date | ………………………………………………………  DD/MM/YY: ………/ …………/ …………. |

**Step 1: Anthropometric Measurements**

| Code | Question | Response |
| --- | --- | --- |
| AM01 | Height measured to the nearest 0.1cm | Height (cm)  └─┴─┴─┘ |
| AM02 | Weight measured to the nearest 0.1 kg  If weight above 120kg code 999.9 | Weight (kg)  └─┴─┴─┴ . └─┴─┴ |
|  | Waist |  |
| AM03 | Waist circumference (to the nearest 0.1cm) | In cm  └─┴─┴─┴ |
| AM04 | Hip Circumference | In cm  └─┴─┴─┴ |
| **Blood Pressure (Reading to be five minutes apart)** | | |
| AM05 | Mid-upper arm circumference | └─┴─┴─┘CM |
| AM06 | Reading 1 | Systolic BP (mnHg)└─┴─┴─┘  Diastolic BP (mnHg)└─┴─┴─┘ |
| AM07 | Reading 2 | Systolic BP (mnHg)└─┴─┴─┘  Diastolic BP (mnHg)└─┴─┴─┘ |
| AM08 | Reading 3 | Systolic BP (mnHg)└─┴─┴─┘  Diastolic BP (mnHg)└─┴─┴─┘ |
| AM09 | Heart Rate count 1 | Heart rate ………………/ minute |
| AM10 | Heart Rate count 2 | Heart rate ………………/ minute |
| AM11 | Heart Rate count 3 | Heart rate ………………/ minute |

**Biochemical Measurements**

| Blood Glucose | | |
| --- | --- | --- |
| Code | Question | Response |
| BI01 | From 12 o’clock last night, have you had anything to eat or drink, other than water? | 1. Yes 2. No   If yes, obtain only one sample for blood sugar. |
| BI02 | Time fasting Blood glucose specimen taken | H H M M  └─┴─┴. └─┴─┴ |
| BI03 | Fasting blood glucose read | Mg/dl  └─┴─┴─┘ |
|  | Blood Lipids – Fasting/ Random | |
| BI04 | Blood specimen taken for Lipids (Cholesterol, Triglycerides, etc.) | 1. Yes 2. No |
| BI05 | Total Cholesterol | Mg/dl  └─┴─┴─┘ |
